# LncRNA SNHG5 promotes the progression of osteosarcoma by sponging the miR-212-3p/SGK3 axis

**DOI:** 10.1186/s12935-018-0641-9

**Published:** 2018-09-18

**Authors:** Cheng Ju, Ruihao Zhou, Jun Sun, Feifei Zhang, Xiaofeng Tang, Kaddie Kwok Chen, Junliang Zhao, Xiaoyong Lan, Shifan Lin, Zhiping Zhang, Xiao-Bin Lv

**Affiliations:** 1 Jiangxi Key Laboratory of Cancer Metastasis and Precision Treatment, The Third Affiliated Hospital of Nanchang University, 128 Xiangshan Northern Road, Nanchang, 330008 Jiangxi People’s Republic of China; 2 Department of Orthopedics, The Third Affiliated Hospital of Nanchang University, 128 Xiangshan Northern Road, Nanchang, 330008 Jiangxi People’s Republic of China; 3 Medical Department of Graduate School, Nanchang University, Nanchang, 330006 Jiangxi People’s Republic of China; 4 First Clinical Department, Medical School of Nanchang University, Nanchang, 330006 Jiangxi People’s Republic of China

**Keywords:** lncRNA SNHG5, miR-212-3p, Osteosarcoma, SGK3, Cell proliferation, Cell invasion and migration

## Abstract

**Background:**

Long non-coding RNA (lncRNA) SNHG5 has been found to play an important role in tumors. Nevertheless, the function and mechanism of lncRNA SNHG5 in osteosarcoma (OS) remains unclear. The purpose of this study was to investigate whether lncRNA SNHG5 can regulate the occurrence and development of OS cells.

**Methods:**

We performed quantitative real time PCR to detect the expression of lncRNA SNHG5 in OS cells. 143B, MG63 (knockdown) and U2OS, U2R (overexpression) cell lines were chosen for the function study of SNHG5. The effect of SNHG5, miR-212-3p, and SGK3 in OS cells was explored by MTT assays, clony formation, flow cytometry, transwell assays, wound healing assays, and cell spreading assays. Quantitative real-time PCR, Western blot analysis and luciferase assays were used to detect the interaction between lncRNA SNHG5 and miR-212-3p.

**Results:**

In this study, knockdown of lncRNA SNHG5 suppressed the growth and metastasis of OS cells, whereas the overexpression of SNHG5 produced an opposite result. Mechanistically, lncRNA SNHG5 functions as a sponger against miR-212-3p and suppresses the miR-212-3p/SGK3 signaling pathway. Introduction of miR-212-3p mimics or inhibitors reverses SNHG5 overexpression or silences the exerted tumor promoting or suppressing effect. In addition, our results showed that the function of SNHG5 can be rescued by miR-212-3p and can regulate the growth and metastasis of OS cells via SGK3, the downstream target of miR-212-3p.

**Conclusions:**

In summary, our study demonstrated that lncRNA SNHG5 can regulate the proliferation and metastasis of OS cells through the miR-212-3p/SGK3 axis. This axis may provide a new target for future clinical treatment.

**Electronic supplementary material:**

The online version of this article (10.1186/s12935-018-0641-9) contains supplementary material, which is available to authorized users.

## Background

Osteosarcoma (OS) is the most common primary malignant bone tumor with the highest incidence in adolescents, often with early metastasis and lung metastases [1, 2]. To date, OS patients regularly suffer from poor clinical prognosis [3–5]. Therefore, it is significant to illustrate the molecular mechanism of OS to help provide new directions and methods for the treatment of OS patients.

About 70%–80% of the genome can be transcribed into RNAs in humans, but only 2%–3% of RNAs can be transcribed to encode proteins [6]. LncRNA refers to RNA larger than 200 nucleotides in length that do not have the ability to code protein [7, 8]. LncRNAs influence various biological processes, including chromatin organization, epigenetic regulation, gene transcription and translation, RNA turnover, and genome defense [9, 10]. In the recent years, increasing studies have found that lncRNAs play an extremely important role in the occurrence and development of tumors [11–13]. At present, a significant number of lncRNAs have been discovered to play an important role in OS. For instance, the upregulation of lncRNA MALAT1, TUG1, HULC, and SNHG12 promote the tumorigenesis of OS [14–17]. Conversely, lncRNA loc285194, TUSC7, and HIF2PUT serve as tumor suppressor genes in OS [18–20].

MicroRNAs (miRNAs) are small non-coding RNA molecules, generally 20–22 nucleotides long, that can mediate the inhibition of transcription and degradation of mRNA via their 3′-untranslated region (3′-UTR) [21]. Several miRNAs have been reported as a suppressor gene or tumor-promoting gene that regulates the proliferation, migration, and invasion of tumors [22, 23]. Existing research shows that the interaction between lncRNA and miRNA is critically important in the progression of cancer. LncRNAs have been also confirmed to regulate the progression of cancers by sponging miRNA [24, 25]. Currently, lncRNA small nucleolar RNA host gene 5 (SNHG5) has been found to act as a tumor-promoting gene in bladder cancer and colorectal cancer [26, 27] and a suppressor gene in gastric cancer [28]. Moreover, SNHG5 can also modulate the progression of cancer by competitively binding to miRNA [29]. These studies show that SNHG5 plays a significant role in a variety of different cancers [26–28, 30–32]. However, the function of SNHG5 in osteosarcoma remains unclear, thus driving us to explore the role of SNHG5 in OS.

In the present study, our results demonstrate that the overexpression of SNHG5 can promote the migration, invasion, and proliferation of OS, whereas knockdown of SNHG5 reduces the migration, invasion, and proliferation of OS cells. Subsequently, our study found that the SNHG5/miR-212-3P/SGK3 axis is critical in the progression of OS.

## Materials and methods

### Cell culture

The human OS cell lines (143B, U2OS, U2R, MG63) were kindly provided by professor Kang [33]. All of the OS cells were cultured in DMEM medium supplemented with 10% fetal bovine serum (FBS, Gibco). 293T cells were cultured in DMEM medium supplemented with 10% FBS (BI). All cells grew in a 37 °C humidified incubator with 5% CO_2_.

### Cell transfection

The overexpression plasmid of SNHG5 (pcDNA-SNHG5) was constructed by inserting the full-length SNHG5 sequences into the pcDNA3.1 vector. The sequence of sh-SNHG5 was cloned into pLKO.1 vector and constructed a SNHG5 knockdown stable cell line for further experiments. Primers of sh-SNHG5 are listed in Table 1; SNHG5 siRNA, SGK3 siRNA, miRNA mimics and inhibitors were purchased from GenePharma Co. Ltd. (SuZhou, China). Lipofecmine 2000 and Lipofectamine^®^ RNAiMAX were used as the transfection reagents according to the manufacturer’s instructions.

**Table 1:** The primer sequences were used in this article

Gene	Sequence
sh-SNHG5-1-F	5′-CCGGGAGGCCAGATTGTCTTGGACTCGAGTCCA AGACAATCTGGCCTCTTTTTTGGTACC-3′
sh-SNHG5-1-R	5′-AATTGGTACCAAAAAAGAGGCCAGATTGTCTTG GACTCGAGTCCAAGACAATCTGGCCTC-3′
sh-SNHG5-2-F	5′-CCGGGCAACGATTTCTGGCTAGTCTCGAGACTAG CCAGAAATCGTTGCTTTTTTGGTACC-3′
sh-SNHG5-2-R	5′-AATTGGTACCAAAAAAGCAACGATTTCTGGCTAG TCTCGAGACTAGCCAGAAATCGTTGC-3′
SNHG5-F	5′-CGCTTGGTTAAAACCTGACACT-3′
SNHG5-R	5′-CCAAGACAATCTGGCCTCTATC-3′
SGK3-F	5′-CCAGGAGTGAGTCTTACAG-3′
SGK3-R	5′-CCAGCCACATTAGGATTA-3′
β-Actin -f	5′-GCCCTGGCACCCAGCACAAT-3′
β-Actin -R	5′-GGAGGGGCCGGACTCGTCAT-3′
ZEB1-f	5′-CAGGCAGATGAAGCAGGATG-3′
ZEB1-R	5′-CAGCAGTGTCTTGTTGTTGTAG-3′
Twist-f	5′-CCAGGTACATCGACTTCCTCTA-3′
Twist-R	5′-CCATCCTCCAGACCGAGAA-3′
FLII-F	5′-CCTCCTACAGCTAGCAGGTTATCAAC-3
FLII-R	5′-GCATGTGCTGGATATATACCTGGCAG-3
BAD-F	5′-ATGTTCCAGATCCCAGAGTTTG-3′
BAD-R	5′-ATGATGGCTGCTGCTGGTT-3′
bcl-xl-F	5′-GCATATCAGAGCTTTGAACAGG-3′
bcl-xl-R	5′-GAAGGAGAAAAAGGCCACAATG-3′
bim-F	5′-AAGGTAATCCTGAAGGCAATCA-3′
bim-R	5′-CTCATAAAGATGAAAAGCGGGG-3′
AIP4-F	5′-GCAGCAGTTTAACCAGAGATTC-3′
AIP4-R	5′-GTGTGTTGTGGTTGACGAAATA-3′
GSK3-β-F	5′-AGGAGAACCCAATGTTTCGTAT-3′
GSK3-β-R	5′-ATCCCCTGGAAATATTGGTTGT-3′

### Transwell assay

Transwell chambers were inserted in 24-well plates. Firstly, cells were washed with serum free media and treated with mitomycin c (10 µg/ml for 30 min). The upper chamber of each well was seeded with 1 × 10^5^ cells with serum-free DMEM medium. DMEM containing 10% fetal bovine serum was added to the lower chamber. To assess cell invasion, 50 μl diluted Matrigel (BD Biosciences, Franklin Lakes, NJ) was added to the upper chamber of the transwell. The 143B, MG63, U2R and U2OS cells were allowed to migrate for 22 h and invade for 24 h. At the specified time, the cells that had migrated or invaded were fixed, stained, and counted.

### MTT assay

MTT assay was used to detect the viabilities of OS cells. Cells (about 1 × 10^4^ cells/well) were placed in 96-well plates and seeded for 24 h, 48 h, 72 h. Subsequently, 4 h before the specified time, MTT solution was added before adding DMSO to dissolve. Finally, cell viability was detected at a wavelength of 450 nm according to the manufacturer’s instructions. Each group was repeated three times to ensure accuracy of the results.

### Cell spreading assay

Cells were centrifuged and resuspended, before allowing to spread on a matrigel-coated plate in a 37 °C humidified incubator with 5% CO_2_. After 1.5 h, cells that did not adhere were washed out with PBS, and the adherent cells were fixed, stained, and counted.

### Flow cytometry

Cell apoptosis was determined using Annexin V-FITC/PI kit (Cat. no: KGA108, Keygen, China). After 48 h the transfection, OS cells were first washed with PBS and resuspended in 500 μl of 1 × binding buffer. Next, 5 μl of Annexin V-FITC and 5 μl of PI were added for 20 min in the dark at room temperature. Finally, flow cytometry (Becton–Dickinson, USA) was performed to detect the number of apoptotic cells according to the Manufacturer’s instructions.

### Clony formation

After transfection of siSNHG5 or pcDNA-SNHG5 for 12 h, OS cells were seeded in 6-well plates at a density of 500 cells per well and maintained for 10 days. Cells were immobilized with paraformaldehyde for 20 min, stained with crystal violet for 30 min and washed with PBS for 3 times. The stained cell colonies were counted.

### Wound healing assays

Wound-healing assays were performed to examine the migratory ability of cells. Firstly, cells were washed with serum free media and treated with mitomycin c (10 µg/ml for 30 min). Transfected cells were cultured in 12-well plates for 24 h. The cell monolayer was scratched using a 20-µl pipette tip, and the cells were cultured for an additional 24 h. The progression of migration was observed and photographed at 0, 12, and 24 h after wounding. The distance between the two edges of the scratch was measured and calculated.

### Real-time PCR

Total RNA was extracted from OS cell lines with TRIzol reagent (Invitrogen, Carlsbad, CA, USA) according to the manufacturer’s instructions. PrimeScript™ RT reagent Kit with gDNA Eraser (TaKaRa, China) was used in order to obtain cDNA. Quantitative real-time PCR (qRT-PCR) was performed using SYBR Select Master Mix for CFX (Invitrogen) and the CFX Connect Real-time PCR system (BioRad) at 95 °C for 15 s, followed by 40 cycles of 95 °C for 5 s, and 60 °C for 34 s. The data were analyzed with the 2^−ΔΔCt^ method. All of primers are listed in Table 1.

### Western blot analysis

Cells were homogenized in RIPA protein lysis buffer supplemented with protease inhibitors on ice for 30 min before centrifuging at 12,000 g for 20 min at 4 °C. The BCA protein assay kit (Pierce, Rockford, IL, USA) was used to measure protein concentration. The total protein extracts were separated by 10% SDS-PAGE and transferred to PVDF membranes (Millipore, USA). The membranes were blocked with 5% non-fat milk for 2 h. Afterwards, the membranes were incubated overnight at 4 °C with the corresponding antibody. Subsequently, the membranes were washed with PBST three times and incubated with secondary antibodies (anti-Rabbit, anti-Mouse) for 2 h. After incubating the anti-Rabbit and anti-Mouse antibodies, the membranes were washed with PBST 3 times and then developed with ECL Western Blotting Substrate. β-actin was used as a control.

### Luciferase assay

The SNHG5 3′-UTRs were constructed into pMIR-reporter plasmids. The SNHG5 and miR-212-3p mimics or NC mimics were co-transfected into 293T cells with Lipofectamine 2000 (Invitrogen), respectively. The Luciferase Reporter Assay System was used for to detecting the luciferase activity.

### Statistical analysis

All of statistical analyses were executed using SPSS 16 software (SPSS, Inc, Chicago, IL, USA). The P-values were calculated using a one-way analysis of variance (ANOVA). A P-value of < 0.05 was considered to indicate a statistically significant result.

## Results

### LncRNA SNHG5 regulates the proliferation and clony formation of osteosarcoma

**Fig. 1:**
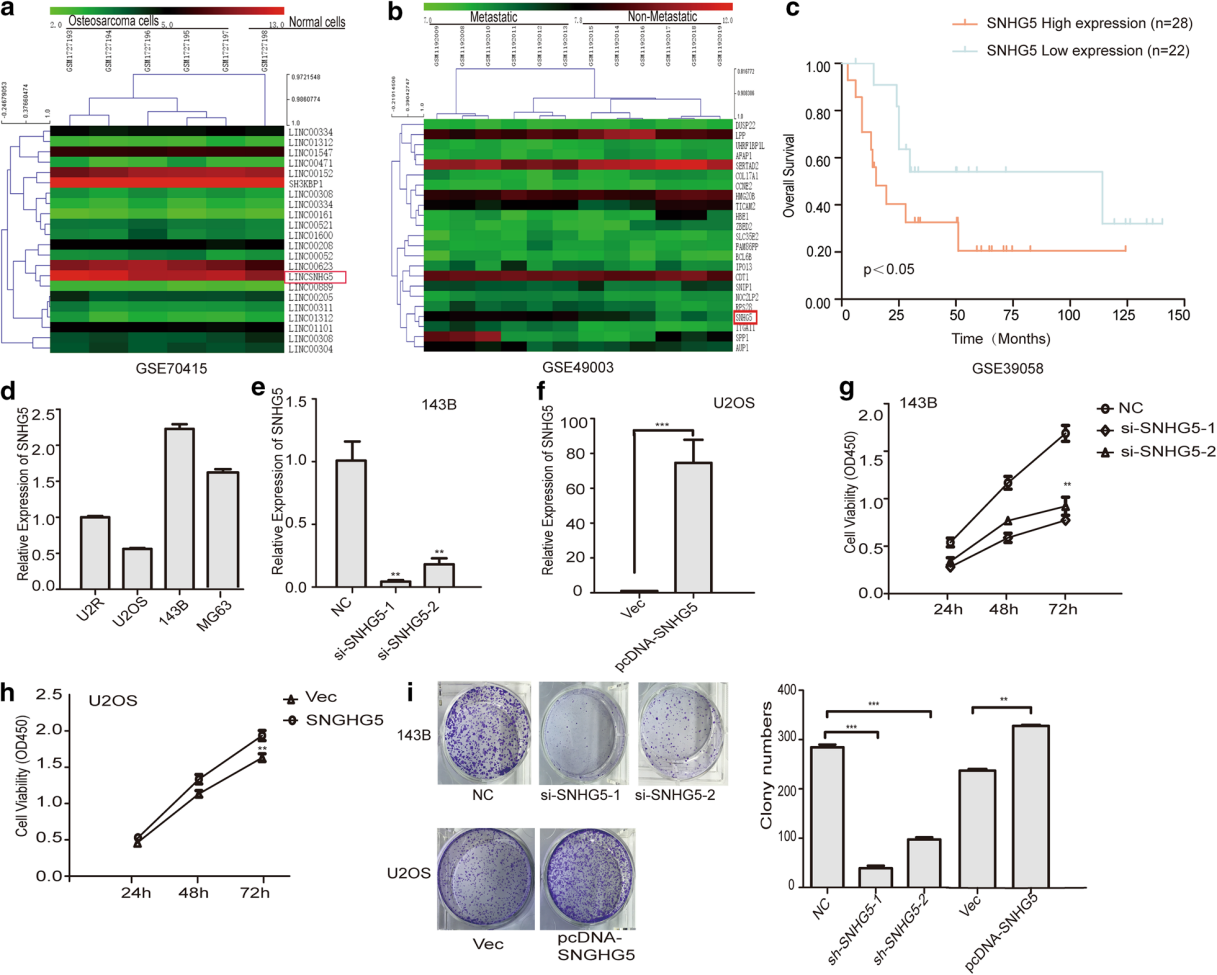
LncRNA SNHG5 regulates the proliferation and colony formation of osteosarcoma. **a**, **b** The microarray data obtained from the GEO database (GSE70415, GSE49003) to investigate the clinical relevance of lncRNA SNHG5 in osteosarcoma. Red or green color in heatmap separately shows high or low expression, according to the color bar in logarithmic scale shown above the heatmap. **c** Kaplan–Meier survival curves for OS patients with SNHG5 expression based on data of GSE39058. **d**–**f** qPCR detected the expression of SNHG5 in OS cells and the successful establishment of the SNHG5 overexpression and knockdown cell models. **g**, **h** The MTT assay shows that cell proliferation is inhibited in the si-SNHG5 group and is enhanced in the SNHG5 overexpression model. **i** The clony numbers decreased in the knockdown model and increased in the overexpression cell models. n = 3, **P < 0.01, ***P < 0.001 vs. control/vector

We analyzed the data from GEO database (https://www.ncbi.nlm.nih.gov/geo/query/acc.cgi?acc=GSE70415, GSE49003, GSE39058) to investigate the clinical relevance of lncRNA SNHG5 in osteosarcoma. Data showed upregulation of lncRNA SNHG5 in osteosarcoma compared to hMSCs (GSE70415, Fig. 1a) and metastatic osteosarcoma compared to non-metastatic (GSE49003 Fig. 1b). Besides, the high expression level of lncRNA SNHG5 is correlated with the patient poor diagnosis (GSE39058, Fig. 1c). To explore the biological function of lncRNA SNHG5 in OS, the expression level of SNHG5 in OS cells (143B, U2OS, U2R, MG63) was detected by real-time PCR (RT-PCR). According to the expression of SNHG5 (Fig. 1d), we transfected siRNAs or the full length of SNHG5 into 143B and U2OS cells, respectively. Efficient silencing of SNHG5 using siRNAs was confirmed using qRT-PCR (Fig. 1e). In addition, Overexpression of SNHG5 increased its RNA level by 70 fold in comparison with the empty vector control (Fig. 1f). The results of the MTT assay and the clony formation assay showed that knockdown of SNHG5 decreased the proliferation of 143B cells, while overexpression of SNHG5 promoted the proliferation of U2OS cells (Fig. 1g–i).

### LncRNA SNHG5 regulates the apoptosis of osteosarcoma cells via caspase pathways

**Fig. 2:**
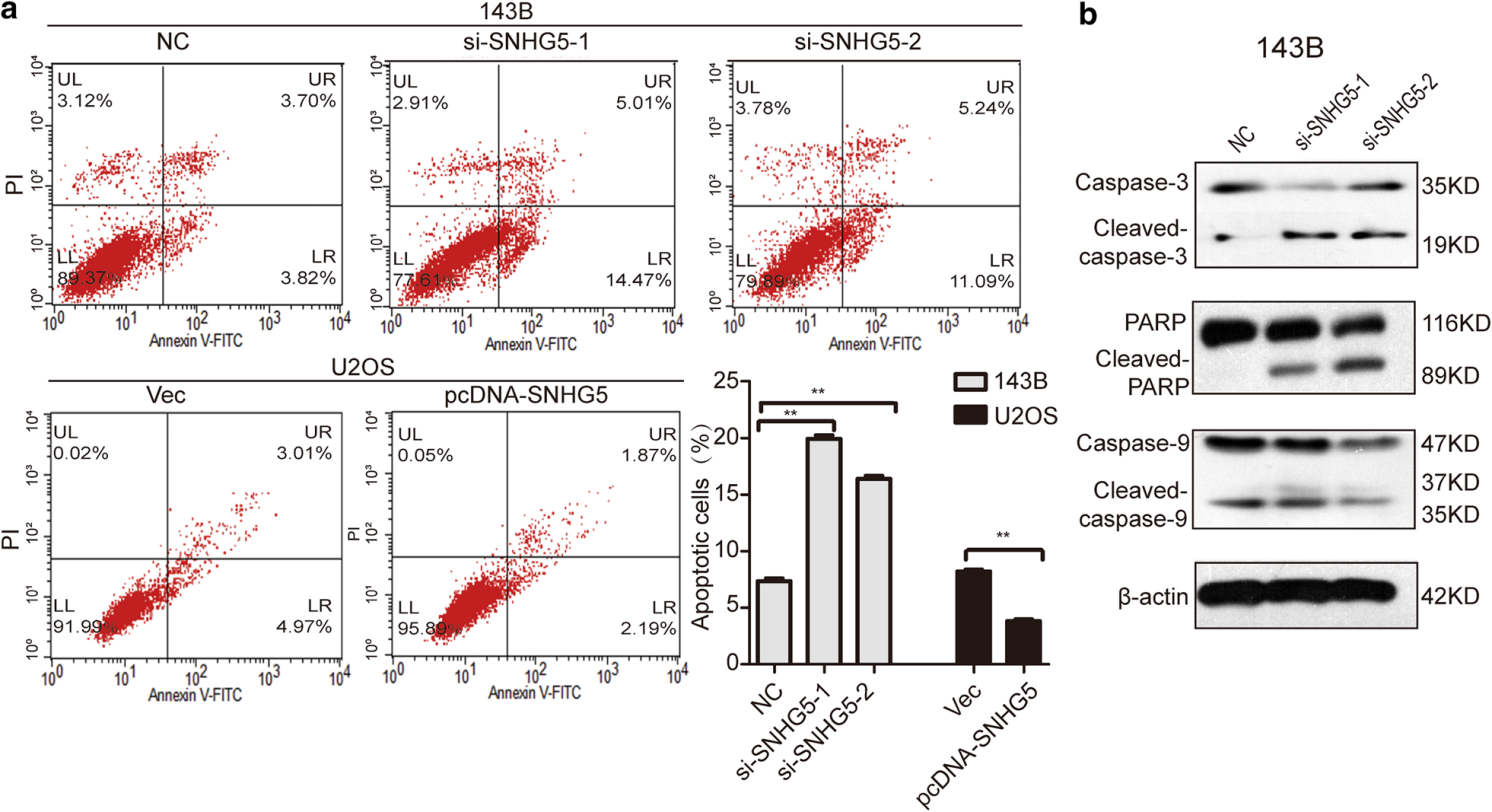
LncRNA SNHG5 regulates the apoptosis of osteosarcoma cells via caspase pathways. **a** Cell apoptosis results were tested by flow cytometry. **b** The protein expression of caspase3, cleaved caspase-3, PARP, and caspase-9 was determined by western-blotting analysis. n = 3, **P < 0.01 compared with NC/Vector

Flow cytometry was utilized to determine the effect of SNHG5 on the apoptosis of OS cells. As shown in Fig. 2a, knockdown of SNHG5 significantly increased the apoptosis of 143B cells. Next, to examine whether knockdown of SNHG5 activated the caspase pathways, western blotting showed that caspase-3, caspase-9, and PARP were activated (Fig. 2b). This suggests that SNHG5 expression can affect the expression of apoptosis and its associated genes and proteins.

### LncRNA SNHG5 regulates the migration and invasion of osteosarcoma cells via the EMT process

**Fig. 3:**
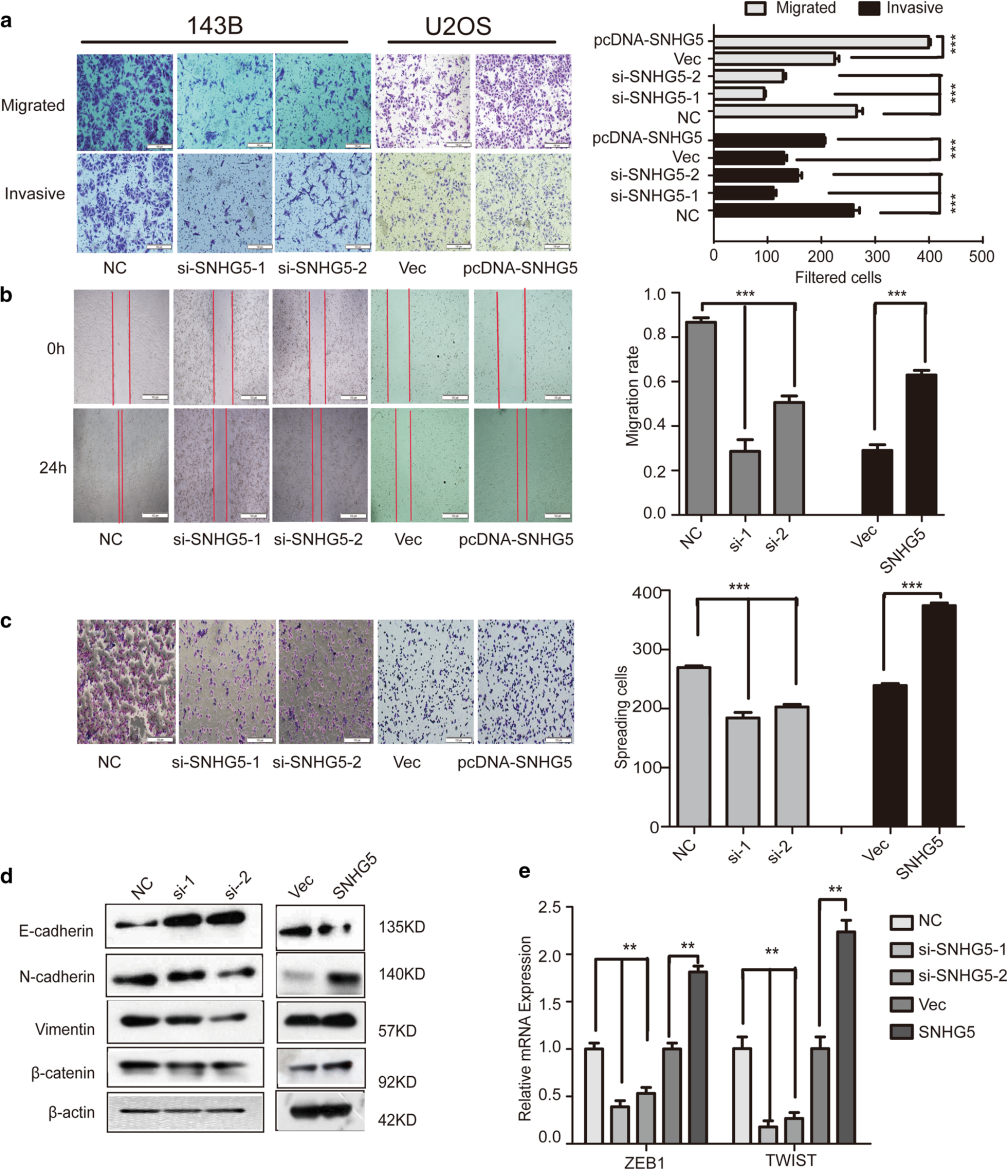
LncRNA SNHG5 regulates the migration and invasion of osteosarcoma cells via the EMT process. 143B and U2OS cells were transfected with si-NC, si-SNHG5-1 si-SNHG5-2, empty vector, or pcDNA-SNHG5 respectively for 48 h. **a** Cell migration and cell invasion assay. **b** Wound healing assay. **c** Cell spread assay were performed. **d** The protein expression of E-cadherin, N-cadherin, Vimentin, and β-catenin was determined by western-blotting analysis. **e** qPCR detected the expression of ZEB1, TWIST. n = 3, ***P < 0.001, compared to NC/Vector

To investigate the role of SNHG5 during cell metastasis and explore the relation of SNHG5 in the EMT process, we performed transwell assays, wound healing assays, and cell spreading assays. The results of the transwell assays and wound healing assays revealed that knockdown of SNHG5 significantly reduced the migration and invasion of 143B cells (Fig. 3a, b). Conversely, overexpression of SNHG5 substantially promoted the migration and invasion of U2OS cells (Fig. 3a, b). Because spreading capacity is one of the important traits contributing to metastasis, cell spreading assays were then conducted with transfected cells. Figure 3c shows that SNHG5 siRNA decreased cell spreading while overexpression of SNHG5 increased it. EMT plays a critical role in the migration and invasion of tumors, as it promotes tumor cell migration and invasion [34, 35]. Thus, we next determined whether EMT related markers were altered in our study. The results showed that the protein expression of N-cadherin, Vimentin, and β-catenin significantly decreased while E-cadherin expression increased when SNHG5 was silenced, in comparison with the si-NC group (Fig. 3d). Compared with the empty vector group, contrasting results were found in U2OS cells after SNHG5 overexpression (Fig. 3d). Besides, the mRNA expression of Twist and ZEB1 transcriptional factors changed paralleled to SNHG5 like other EMT markers (Fig. 3e). Thus, these results demonstrated that lncRNA SNHG5 was involved in the EMT process and knockdown of SNHG5 inhibits the EMT process in OS cells. Besides, we confirmed the function of lncRNA SNHG5 in other two OS cells via MTT, clony formation, flow cytometry, transwell assay (MG63, U2R). The results showed LncRNA SNHG5 regulated the proliferation, migration and invasion of osteosarcoma cells (Additional file 1: Figure S1).

### LncRNA SNHG5 acted as a direct target of the miR-212-3p

**Fig. 4:**
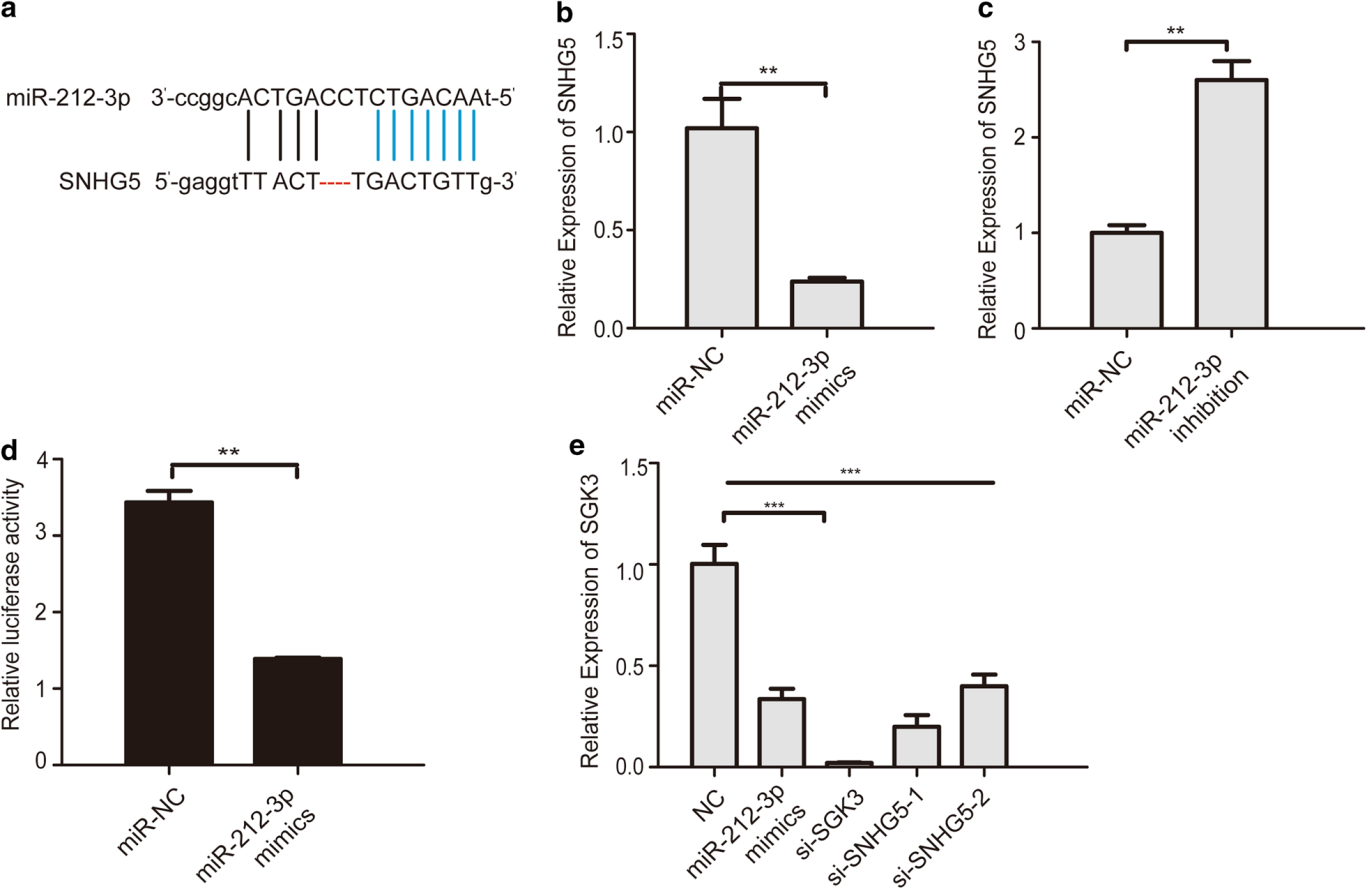
LncRNA SNHG5 acted as a direct target of the miR-212-3p. **a** Bioinformatics analysis identified a potential miR-212-3p binding site of SNHG5. **b**, **c** qPCR detected the expression of SNHG5 after transfection of miR-212-3p mimics and inhibitors. **d** Luciferase reporter assay detected the luciferase activity of cells co-transfected with miR-335-5p mimics or miR-NC and SNHG5. **e** qPCR detected the expression of SGK3 after transfection of mimics, si-SGK3, si-SNHG5. n = 3, **P < 0.01, compared to miR-NC

The ceRNA hypothesis presumes that specific lncRNA can actas sinks for pools of active miRNAs [36, 37], such as Long noncoding RNA GAPLINC promotes gastric cancer cell proliferation by acting as a molecular sponge of miR-378 to modulate MAPK1 expression [38], Long noncoding RNA DANCR promotes colorectal cancer proliferation and metastasis via miR-577 sponging [39] To further explore the mechanism of SNHG5 in osteosarcoma, an online bioinformatics software, Starbase (http://starbase.sysu.edu.cn/), was used to predict the target sites of lncRNA SNHG5 and miR-212-3p miRNA (Fig. 4a). Simply, we inserted the gene symbol of lncRNA SNHG5 into database of Starbase v2.0 in order to find possible target miRNAs and the system showed that lncRNA SNHG5 may interact with miR-212-3p. RT-PCR results reveal that the expression of SNHG5 reduced after transfection of miR-212-3p mimics (Fig. 4b). Conversely, knockdown of miR-212-3p significantly increased the expression of SNHG5 (Fig. 4c). Subsequently, we constructed plasmids expressing a chimera gene SNHG5-luciferase. Then we transfected cells with control miRNAs (miR-NC) or with mir-212-3p mimic. This latter one reduced the luciferase expression (Fig. 4d). SGK3 has been reported to be an important tumor-promoting gene and the target gene of miR-212-3p in glioblastoma cells [40–42]. Q-PCR results showed that transfecting miR-212-3p mimics, si-SGK3 and si-SNHG5 strongly reduced the expression of SGK3 (Fig. 4e). The above results showed that SNHG5 might act as a direct target of miR-212-3p.

### LncRNA SNHG5 as a molecular sponge of miR-212-3p to further promote the growth and migration of OS

**Fig. 5:**
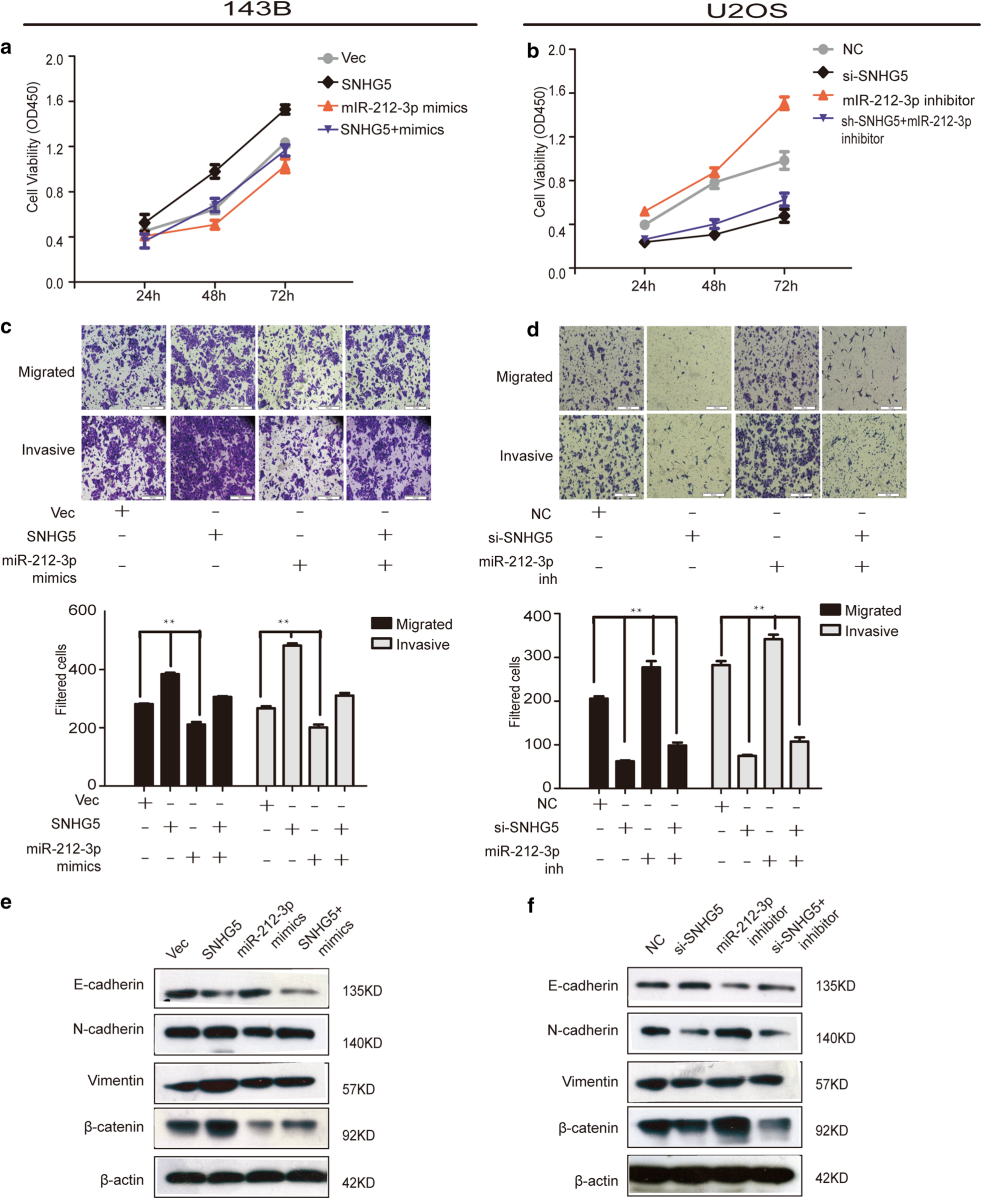
LncRNA SNHG5 as a molecular sponge of miR-212-3p to further promote the growth and migration of OS. **a**–**d** MTT and transwell assays were performed to detect the cell growth, migration, and invasion abilities after SNHG5 overexpression and knockdown, which was rescued by miR-212-3p mimic and inhibitor treatment, **e**, **f** The protein expression of E-cadherin, N-cadherin, Vimentin, and β-catenin was determined by western-blotting analysis. n = 3, **P < 0.05, compared to NC/Vector

Next, we explored the interaction of SNHG5 and miR-212-3p in OS cell growth, migration, and invasion. The results of MTT and transwell assays showed that overexpression of miR-212-3p could reduce the growth, invasion, and migration of pcDNA-SNHG5 (Fig. 5a, b). Conversely, knockdown of miR-212-3p significantly increased the cell proliferation, invasion, and migration abilities of OS cells. In fact, this increase could be restored by co-transfecting si-SNHG5-mixture with miR-212-3p inhibitors (Fig. 5c, d). In addition, the protein expression of EMT markers in two rescue assays was also verified (Fig. 5e, f).

### LncRNA SNHG5/miR-212-3p/SGK3 axis regulate the progression of osteosarcoma

**Fig. 6:**
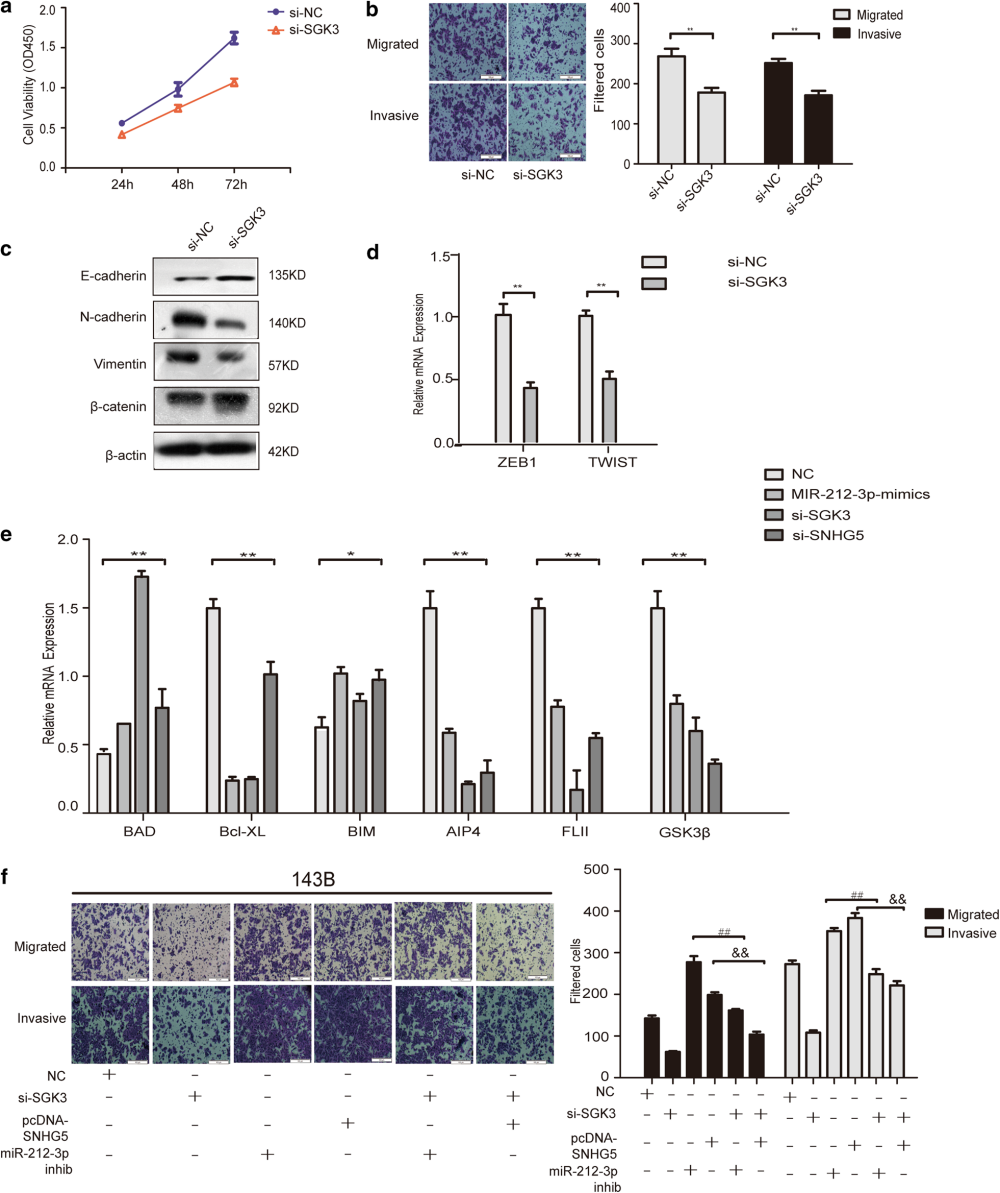
LncRNA SNHG5/miR-212-3p/SGK3 axis regulate the progression of osteosarcoma. **a**, **b** Transwell and MTT assays were performed to detect cell growth, migration, and invasion abilities after SGK3 silencing. **c** The protein expression of E-cadherin, N-cadherin, Vimentin and β-cantenin were determined by western-blotting analysis. **d** qPCR detected the expression of ZEB1, TWIST. **e** qPCR detected the expression of Bad, Bim, Bcl-xl, AIP4, FLII, GSK3β after transfection of mimics, si-SGK3, si-SNHG5. **f** Transwell assays were performed to detect the cell migration and invasion abilities after SGK3 knockdown, which was rescued by pcDNA-SNHG5 and miR-212-3p inhibitor treatment. n = 3, **P < 0.01, compared to NC, ## P < 0.01, inhibition vs. si-SGK3+inhibition, && P < 0.01, pcDNA-SNHG5 vs. si-SGK3+pcDNA-SNHG5

**Fig. 7:**
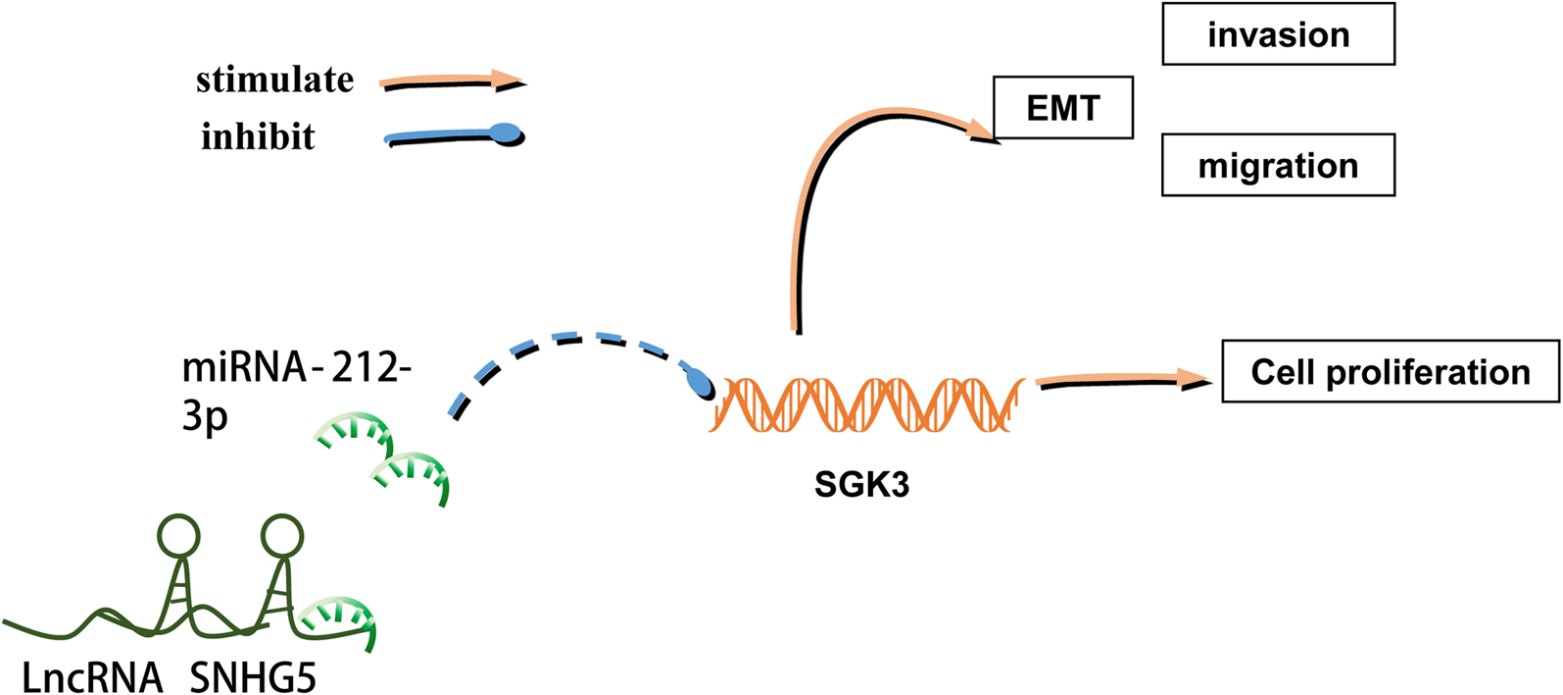
Pathway diagram of LncRNA SNHG5/miR-212-3p/SGK3 axis

Recently, SGK3 has been reported to be an important tumor-promoting gene [40, 41]. Moreover, SGK3 has been recently published as the target gene of miR-212-3p in glioblastoma cells [42]. However, the function of SGK3 in OS has not been confirmed. Knockdown of SGK3 decreased the migration, invasion and growth of OS (Fig. 6a, b). Furthermore, we found that the expression of our above mentioned EMT markers significantly decreased when SGK3 was silenced, while E-cadherin expression was increased—which was consistent with SNHG5 expression (Fig. 6c, d). The above results showed that lncRNA SNHG5 might regulate the progression of OS through the miR-212-3p/SGK3 axis. Expression of other apoptosis associated genes were performed especially as previous researchers have been shown to change with SGK3 knockdown such as Bad, Bim, Bcl-xL [43–45]. The results showed that the expression of SGK3 was consistent with Bcl-xl while opposite to Bad and Bim (Fig. 6e). Then we detected the expression of SGK3’s direct targets such as AIP4, FLII, GSK3β [43–45]. Results showed that the expression of these targets was consistent with SGK3 (Fig. 6e). The results of transwell assays showed that overexpression of SNHG5 and inhibition of miR-212-3p could extenuate the reduction of invasion, and migration of si-SGK3 (Fig. 6f), which showed LncRNA SNHG5/miR-212-3p/SGK3 axis regulate the progression of osteosarcoma. Above all, we illustrated the pathway diagram of LncRNA SNHG5 / miR-212-3p/SGK3 axis in osteosarcoma (Fig. 7).

## Discussion

OS is the most common malignant bone tumor in adolescents. Between 70 and 80% of patients are 10 to 25 years old, with an annual incidence of 1–3/100,000 [46, 47]. Currently, the 5-year survival rate of OS patients is still in the range of 60% to 70% due to the unclear pathogenesis of OS [48]. Thus, it is vital that the molecular mechanism of OS is evident to improve treatment.

Many previous studies have elaborated on the functions of lncRNA SNHG5 in detail. For example, SNHG5 promotes colorectal cancer cell survival by counteracting STAU1-mediated mRNA destabilization [27]. SNHG5 is also associated with poor prognosis of bladder cancer, and promotes bladder cancer cell proliferation through targeting P27 [26]. Long non-coding RNA SNHG5 also suppresses gastric cancer progression by trapping MTA2 in the cytosol [28], in addition to being a new biomarker in malignant melanoma [31]. These studies demonstrate the importance of SNHG5 in cancers. In our study, we found that overexpression of SNHG5 promoted OS cell growth. Yet, knockdown of SNHG5 inhibited OS cell proliferation and induced apoptosis through activating cleaved-caspase-3, cleaved-caspase-9, and cleaved-PARP. However, it has been demonstrated that mesenchymal stromal cells, from which osteosarcoma cells originate. Interestingly, Alessio et al. [49] found cells are prone to senescence rather than apoptosis even after high exogenous stress. This is also worth further exploring. In addition, EMT plays a critical role in the migration and invasion of tumors, as it promotes tumor cell migration and invasion [34, 35]. E-cadherin acts as an epithelial marker that is lowly expressed in tumors, whereas vimentin and β-catenin are interstitial markers highly expressed in tumors [50]. As key proteins of EMT, upregulation of E-Cadherin and downregulation of vimentin and β-catenin are considered characteristic expressions of EMT. In the present study, western blotting revealed that the knockdown of SNHG5 increased the expression of E-cadherin and reduced the expression of Vimentin and β-catenin in OS cells. The overexpression of SNHG5 produced an opposite result.

In recent years, the competing endogenous RNA (ceRNA) hypothesis has been extensive promoted, and some studies have confirmed the interaction of lncRNA and miRNA in multiple cancers [24, 25]. In previous studies, lncRNA SNHG5 was found to be able to interact with miRNA. For example, long non-coding RNA SNHG5 regulates gefitinib resistance in lung adenocarcinoma cells by targeting the miR-377/CASP1 axis [32]. The lncRNA SNHG5/miR-32 axis regulates gastric cancer cell proliferation and migration by targeting KLF4 [29], in addition to regulating imatinib resistance in chronic myeloid leukemia via MiR-205-5p [30]. To examine whether existing miRNA can bind to lncRNA SNHG5 in OS cells, we used an online bioinformatics software, Starbase, to predict the sites of lncRNA SNHG5 and miRNA, in which miR-212-3p was chosen for further study. Interestingly, we found that researcher [51] evaluated the expression of miR-212-3p in OS tissues by RT-qPCR with the result showing a substantial decrease of miR-212-3p expression in OS tissues compared to that in normal tissues, which was consistent with our results.

Subsequently, luciferase report assays and RT-PCR results showed that miR-212-3p can be combined with SNHG5 to reduce the activity of SNHG5. However, rescue assays revealed that miR-212-3p mimics suppress the growth and metastasis of pcDNA-SNHG5, and that its the restoration can be induced by co-transfecting the si-SNHG5-mixture with miR-212-3p inhibitors.

SGK3 has been found in others cancers as a carcinogenic gene [42, 52–55]. A recent report revealed that SGK3 plays a vital role in glioblastoma as the target gene of miR-212-3p [42]. To explore whether SGK3 could also promote the proliferation and metastasis of OS cells, we performed a series of assays to detect the function of SGK3. MTT assays showed that knockdown of SGK3 reduced the growth of OS cells. Transwell assays and western blotting indicated that knockdown of SGK3 suppressed the migration and invasion of OS cells via the EMT process.

## Conclusions

Overall, our current study demonstrated SNHG5 is involved in the regulation of OS cell progression. What is more meaningful is that SNHG5 regulated the proliferation, migration, and invasion of OS cells via the miR-212-3p/SGK3 axis. Consequently, these conclusions showed that lncRNA SNHG5 plays as a critical role in OS tumorigenesis—indicating that SNHG5 may be a potential therapeutic target for osteosarcoma.

## Additional file

**Additional file 1: Figure S1.** The function study of LncRNA SNHG5 in MG63 and U2R OS cell.

## Abbreviations

lncRNA: long non-coding RNA
SNHG5: small nucleolar RNA host gene 5
OS: osteosarcoma
qRT-PCR: quantificational real-time quantitative polymerase chain reaction
