# Supplementary figures and images for: LncRNA SNHG5 promotes the progression of osteosarcoma by sponging the miR-212-3p/SGK3 axis
Source: Cancer Cell Int. 2018 Sep 18;18:141. doi: 10.1186/s12935-018-0641-9 (PMC6145323; doi:10.1186/s12935-018-0641-9)

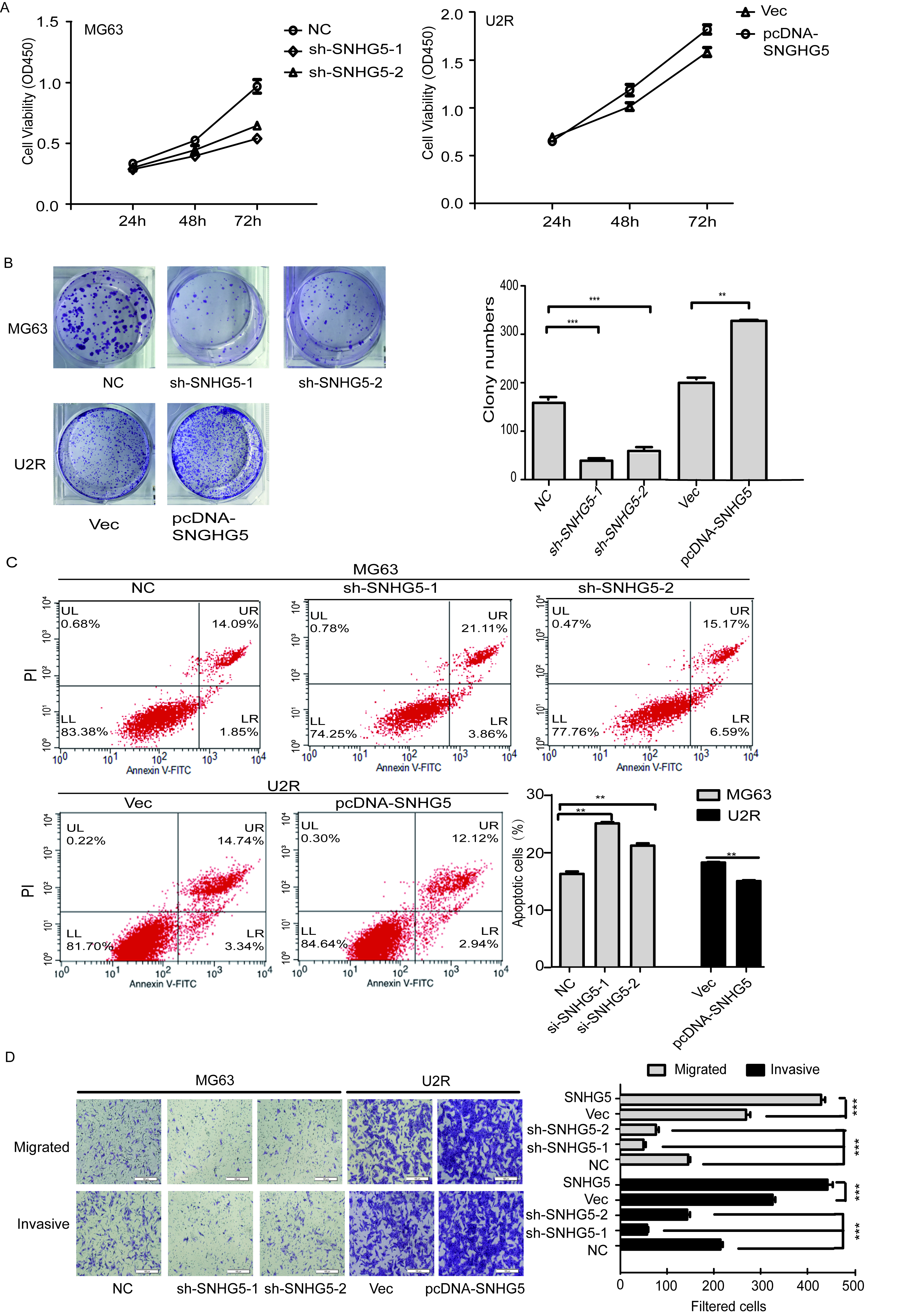

Supplement: Supplementary file 1 — Additional file 1: Figure S1. The function study of LncRNA SNHG5 in MG63 and U2R OS cell. [file 12935_2018_641_MOESM1_ESM.tif]
